# Mycomerge: Fabrication of Mycelium-Based Natural Fiber Reinforced Composites on a Rattan Framework

**DOI:** 10.3390/biomimetics7020042

**Published:** 2022-04-08

**Authors:** Mai Thi Nguyen, Daniela Solueva, Evgenia Spyridonos, Hanaa Dahy

**Affiliations:** 1 Faculty of Architecture and Urban Planning, University of Stuttgart, Keplerstrasse 11, 70174 Stuttgart, Germany; st163415@stud.uni-stuttgart.de (M.T.N.); st161479@stud.uni-stuttgart.de (D.S.); 2 BioMat Department of Bio-Based Materials and Materials Cycles in Architecture, Institute of Building Structures and Structural Design (ITKE), University of Stuttgart, Keplerstrasse 11, 70174 Stuttgart, Germany; hanaa.dahy@itke.uni-stuttgart.de; 3 Department of Architecture (FEDA), Faculty of Engineering, Ain Shams University, Cairo 11517, Egypt; 4 Department of Planning, Technical Faculty of IT & Design, Aalborg University, 2450 Copenhagen, Denmark

**Keywords:** bio-based materials, mycelium, mycelium-based composites, natural fiber reinforced polymers, NFRP, growing materials, rattan, lightweight structure

## Abstract

There is an essential need for a change in the way we build our physical environment. To prevent our ecosystems from collapsing, raising awareness of already available bio-based materials is vital. Mycelium, a living fungal organism, has the potential to replace conventional materials, having the ability to act as a binding agent of various natural fibers, such as hemp, flax, or other agricultural waste products. This study aims to showcase mycelium’s load-bearing capacities when reinforced with bio-based materials and specifically natural fibers, in an alternative merging design approach. Counteracting the usual fabrication techniques, the proposed design method aims to guide mycelium’s growth on a natural rattan framework that serves as a supportive structure for the mycelium substrate and its fiber reinforcement. The rattan skeleton is integrated into the finished composite product, where both components merge, forming a fully biodegradable unit. Using digital form-finding tools, the geometry of a compressive structure is computed. The occurring multi-layer biobased component can support a load beyond 20 times its own weight. An initial physical prototype in furniture scale is realized. Further applications in architectural scale are studied and proposed.

## 1. Introduction

### 1.1. Relevance

Since the linkage between human consumption behavior and the rapidly increasing global warming becomes evident, life in the 21st century faces an ongoing environmental crisis. The use of conventional building materials in the global industry impacts largely on climate change by destructing more than 45 percent of global resources and emitting up to 40 percent of the energy-related carbon dioxide into the atmosphere [1,2]. Within an ever-growing society, there is and will be a constant need for materials, and consumption prevention is not the optimal choice. Awareness of already existing alternative systems is crucial for achieving sustainability. Waste can only be repurposed as a new resource if the majority of building components can be disassembled and returned to their original material cycles separately [3,4]. Fungal substrates are considered waste products and thus, can be used as compost and in a range of other applications [5]. By decreasing the cradle-to-gate manufacturing in the building sector, construction processes could be optimized to meet the social, ecologic, and economic values of the future generation. While the composting process was absent during the non-regenerative production line of the 18th and 19th centuries, at the present time, reorientation processes take place towards the cultivation of natural resources by breeding, raising, or growing materials. With sufficient improvement of the current fabrication methods, which are biologically driven and technologically supported, designing and manufacturing sustainable objects are goals within reach [3,6,7].

### 1.2. Recyclability of Composites

With an increasing demand for lightweight and durable building materials, fiber-reinforced polymer composites are considered a reliable alternative to conventional building materials such as concrete and steel. Determined by their components and fabrication methods, the structural and functional performance of fiber-reinforced polymers (FRC) can be adjusted to match the preferred application. Natural fiber-reinforced polymer (NFRP) composites consist of a high-strength reinforcement and a high-ductility matrix. Cellulosic fibers are highly sustainable and commonly used as reinforcement in composites. They are commonly agricultural residues; hence they enhance the ecological role of renewable resources, can be found in nature, are non-toxic, renewable, cost-effective, and allow bonding with different matrices. Bio-based composites with mineral and petrochemical matrices are widely used. However, their full biodegradability is costly to achieve due to the complex separation of the composites into their initial components, causing limited end-of-life options. Recyclability of composites with bio-based matrices is also limited, as degradation can only take place in specific industrial composting conditions [4,6,8]. Alternatively, mycelium-based matrices are organic matter and fully biodegradable, fulfilling the requirements of the circular material life cycle [8,9]. Since the main constituents of mycelium composites are fibrous substrates, lignocellulosic agricultural or forestry by-products and wastes such as straw and hemp, or porous substrate, e.g., sawdust, the costs of mycelium composites are low and enable waste upcycling [10,11]. The results of the first methods for the disintegration of mycelium-based composites (Ganoderma resinaceum and hemp fibers) in soil have strengthened their biodegradability, with a maximum weight loss of 43% after 16 weeks [12].

### 1.3. Mycelium Based Composites

Mycelium is the root of fungi, building large thread-like networks, which are made of individual hyphae. Hyphae grow from mycelium fungal strain spores and consume feedstock containing carbon and nitrogen [13]. To create mycelium-based composites, fast and robust colonization of the substrate is required. Among the numerous subordinates of fungi, Dikarya build large and complex structures. These fungi have two special characteristics: Septa—transverse cell wall opening which can close—decreases damage caused to the colony by a rupture; and Anastomosis—the ability of two hyphae fusing together to build large networks and distribute nutrients from high to low concentrated areas. In the presence of hosting materials from agricultural waste products such as hemp or flax, mycelium merges with its environment and absorbs its host. Colonization and growth are highly dependent on the amount of cellulose in the given hosting material, as the nutrition of fungi consists of glucose. Mycelium can break down cellulose into glucose, which means that a high cellulose environment can improve its growth. Apart from compatibility, some natural fibers offer additional protection by a waxy outer layer, preventing contamination by other microorganisms. Generally, the hosting material must be sterilized through the processes of pasteurization, hydrogen-peroxide treatment, and natural composting [8]. The substrate can then be inoculated with the preferred fungal species. There are crucial growing conditions for successful cultivation, including low light, high humidity, medium temperature, and access to oxygen. After sufficient growth, the growing phase can be interrupted or stopped by exposing the cultivated composite to high temperatures over 80 degrees [8,9,13].

### 1.4. Previous Studies

Mycelium-based biocomposites are perceived as a sustainable and competitively performing material alternative in several application fields, including thermal and acoustic insulation, or as a replacement to standard expanded polystyrene packaging [10]. Because of mycelium’s high compressive mechanical properties, previous studies have focused on compression-only structural applications. Further applications of mycelium in design and construction have also been a topic of further research studies [14].

A study carried out by Jiang (2017) [15] examines the performance of mycelium-bound sandwich composites by measuring the flexural stiffness of the composites’ core and skin layers. During this experiment, a discontinuous composite core is placed in between two skin layers of continuous and randomly oriented cellulosic fibers. Interfacial bonding between the core composite and the outer binding layers was possible because of mycelium’s ability to grow through the fiber matrix and effectively bind with the fibers. The compressive properties of the composite are mainly dependent on the core stiffness and can be improved through highly cellulosic skin materials [10].

**Figure 1:**
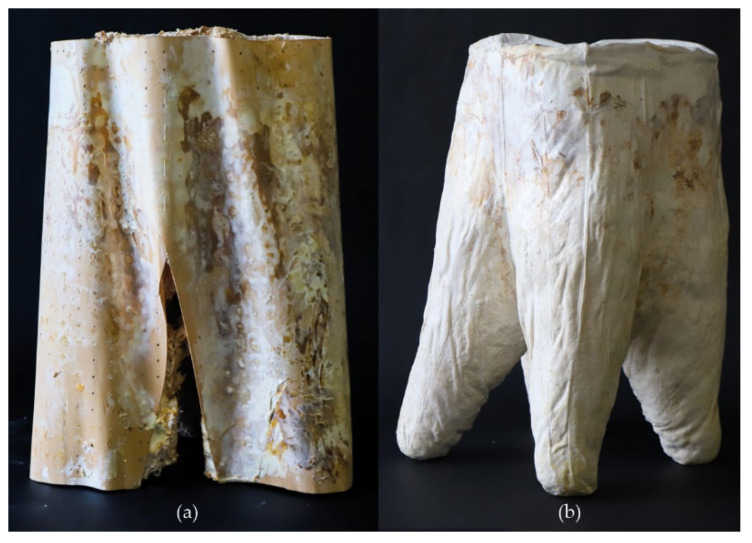
Mycelium-based prototypes: (**a**) using a timber veneer mold, and (**b**) using cotton fabric mold, by F. Milano, K. Antorveza, L. Kiesewetter, G. Lochnicki, Materials Matter Lab IV, 2020.

Two mycelium composite prototypes were developed during the Material Matter Lab IV at the BioMat Department in the ITKE Institute of the University of Stuttgart. This is a seminar practicing validation through small-scale structural demonstrators in the form of chairs and stools [16]. A timber veneer mold (Figure 1a) and a soft cotton fabric mold (Figure 1b) were used to develop the two alternatives. The timber mold bends because of the moisture being held throughout the entire growth process, interfering with the overall shape and causing separation. In addition, due to the outer skin’s density, there is insufficient ventilation, which leads to mold contamination. Separation and inconsistent formability occurred in the cotton fabric option prototype, just as in the previous experiment, because of the fabric’s stretchability. In industrial treatment methods such as bleaching, nutrition loss occurs in natural cellulose material, making it less suitable for the mycelium to grow on.

## 2. Materials and Methods

### 2.1. Workflow

**Figure 2:**
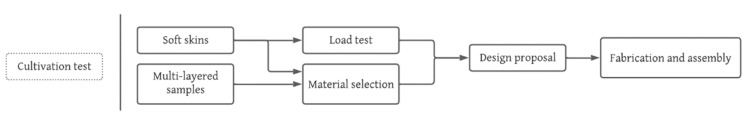
Bottom-Up workflow diagram.

After obtaining sufficient knowledge about the environmental preferences and growing behavior of mycelium, the initial step of the practical process is the proper cultivation of the organism. The aim is to prevent creating an external mold that will have to be discarded. The bonding qualities of mycelium and outer skin materials are investigated through small-scale samples. Digital form-finding and optimization tools, specifically Rhino Vault 2, a plugin for Rhino McNeel that concentrates on funicular form-finding, are used to calculate design possibilities. A rattan framework is used as a supporting skeleton to enable fabrication without the usage of an external mold while also being able to form double-curved surfaces. A workflow diagram in Figure 2 presents the basic steps of this bottom-up process.

### 2.2. Cultivation of Homegrown Substrate

**Figure 3:**
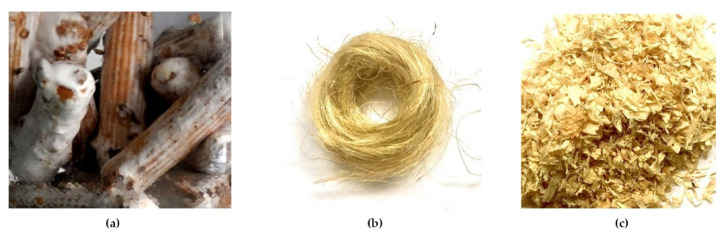
Mycelium-infused wood plugs (**a**), hosting materials: hemp fibers (**b**), wood chips (**c**).

**Figure 4:**
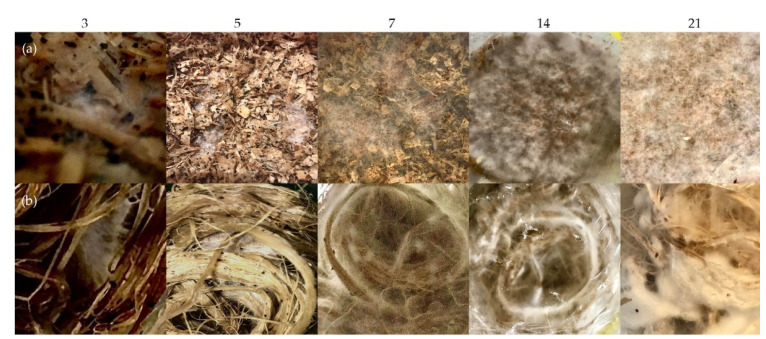
Growth process in days: (**a**) wood chips, (**b**) hemp fibers.

To inoculate the substrate, wood plugs already infused with *Pleurotus ostreatus* cultures are utilized. Short, chopped fibers such as wood chips and long continuous fibers such as hemp are compared as a hosting environment (Figure 3). The hosting materials are sterilized via pasteurization. Then, the sterilized materials must cool down to a temperature of 28 °C before adding nutrients and the mycelium-infused wood plugs. The growing period takes place at an ambient temperature of approximately 20–25 °C over the course of three weeks (Figure 4).

### 2.3. Compatibility with Skin Materials

**Figure 5:**
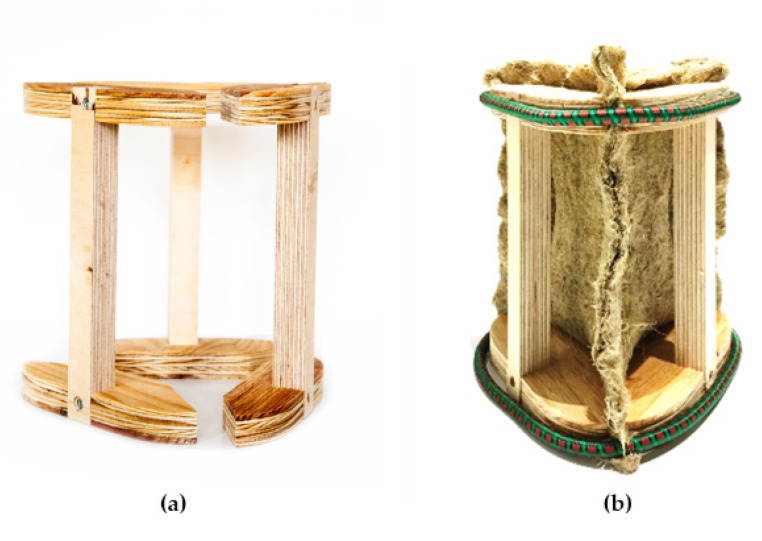
(**a**) Wooden framework, (**b**) hemp mat.

The pre-grown substrate was purchased from the market due to the lack of a sterile environment throughout this study, as well as the time constraints imposed by the long growing period required. The substrate’s merging capabilities are examined using three natural fiber materials: continuous bidirectional woven jute fabric, discontinuous randomly oriented compressed hemp sheets, and a continuous unidirectional hemp rope knitted outer layer. An easily detachable framework is necessary to keep the sterilized soft textiles in position (Figure 5). Wooden frames are CNC cut and then assembled. After the material growth is completed, each component of the framework can be detached and reused.

### 2.4. Results of Growth on Skin Materials

#### 2.4.1. Hemp Sheets

**Figure 6:**
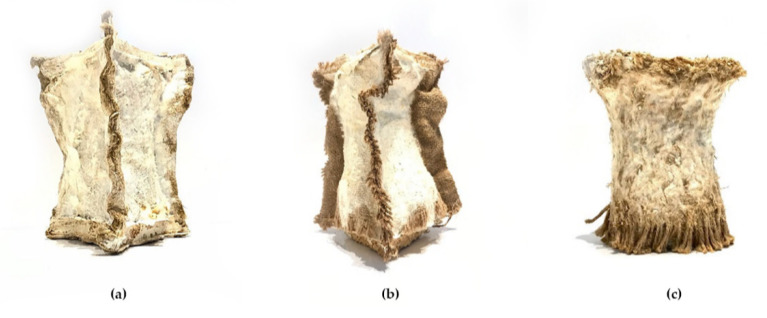
Results of grown on different skin materials: (**a**) hemp sheets, (**b**) jute sheets, (**c**) knitted hemp rope.

The first sample contains mycelium substrate, compressed into a soft mold of randomly oriented hemp fibers (Figure 6a). Due to the high density of the hemp sheets, water absorption levels are high and ensure constant moisture levels throughout the growth process. Because of that, the mycelium grew beyond the geometrical restrictions of the soft mold and along the outer edges of the hemp sheets. The high growth density prevents separation during the shrinking process and results in the stiffest sample and most successful binding outcome. The concept of a “soft mold” suggests the use of a natural frame that is integrated during the fabrication process and stays embedded in the end-product. The outcome of this initial test was successful, resulting in new ideas for alternate molding methods.

#### 2.4.2. Jute Sheets

In the second experiment, pre-woven bidirectional jute sheets are used as a skin alternative (Figure 6b). The low thickness and density of the fibers do not contribute to containing sufficient moisture levels, which results in the sample drying before enough growth is achieved. Consequently, uneven shrinkage and separation between substrate and skin layer occurred.

#### 2.4.3. Knitted Hemp Rope

Throughout the third experiment, knitted hemp rope is used as an alternate outer skin (Figure 6c). While compressing the mycelium substrate into the mold, the too loosely knitted skin resulted in a deformed overall shape. Similar moisture deficiency as in the previous observation occurs. The sample’s final state is less rigid and hardly successful, due to the uneven distribution of the substrate.

### 2.5. Results on the Growth of Multi-Layer Samples

Based on the successful growing outcome of the first sample (Section 2.4.1), two further experiments with hemp were carried out.

#### 2.5.1. Hemp Sheet Sandwich

**Figure 7:**
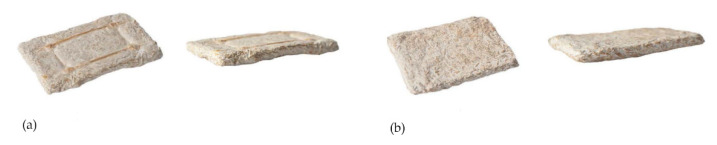
(**a**) Results of multilayer composite, (**b**) hemp mat sandwich.

As an alternative to filling up a voluminous mold, in this experiment, the mycelium substrate is pressed between two hemp mat layers to form a thin and rigid sandwich element. As in the above-described example, the density of the hemp fibers ensures a constantly moist growth environment. No separation between substrate and hemp sheets is visible. This sandwich results in the stiffest sample (Figure 7b).

#### 2.5.2. Multilayer Composite: Rattan, Loose Hemp Fibers, Mycelium Substrate with Chopped Hemp Fibers

In the final material test, loose hemp fibers act as a substitute for the mechanically compressed hemp sheets, and rattan reinforcement is introduced in between the mycelium substrate. Due to the greater airflow between the randomly oriented loose hemp fibers, there is proportionally more space for the mycelium to spread, still resulting in a stiff sample but also exhibiting higher elasticity. Rattan acts as an integral structural reinforcement, as it successfully merges with the mycelium. This compatibility leads to a significant increase in the overall stiffness (Figure 7a).

### 2.6. Form-Finding

**Figure 8:**
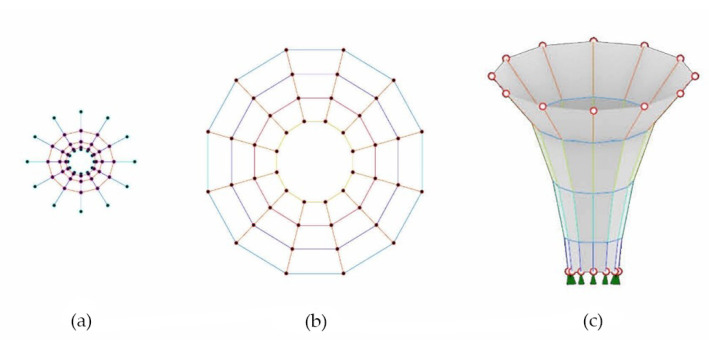
(**a**,**b**) Form and Force Diagram, (**c**) Thrust Diagram.

Based on the findings of Section 2.4 and Section 2.5, considering the volume of small-scale samples is sufficient to hold a person’s weight of approximately 80 kilos, a prototype in the form of a stool, named Mycomerge, is designed and built. The geometry is developed through form-finding procedures using Rhino Vault 2. The digital form-generating methods are used to create the entire geometry as well as for basic optimization of the structure [17]. The structure’s skeleton, in this case, the rattan framework, is first generated, starting with single lines and the core of the geometry. Then three-dimensional surfaces are integrated to form the rest of the shape (Figure 8). The main parameters of the computational model are associated with the grid density for the skeleton and the overall dimensions. A “funnel-shaped” structure is generated, which is only supported centrally, forming a canopy that cantilevers without the need for additional supports at its edge [18,19]. In this case, the resulting canopy acts as the seating area, with the center of gravity meeting at the central support. The purpose of this design is to maximize material efficiency while achieving the appropriate load-bearing capacities using the least amount of material. The stool is designed with a seating height of 45 cm to provide comfortable seating. The funnel shell of the resulting Thrust Diagram (Figure 8) is used for developing the arrangement of the rattan skeleton, which serves as an integrated structural element on which the fibers and mycelium substrate are placed.

### 2.7. Prototyping

**Figure 9:**
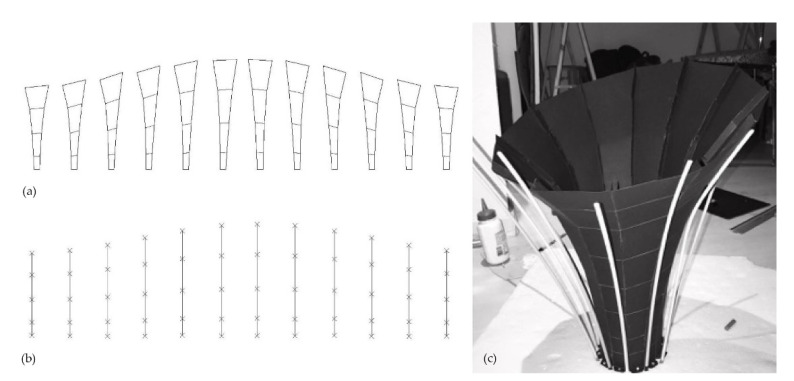
(**a**) Layout for paper strips, (**b**) goal lengths, (**c**) assembled paper model.

A full-scale paper model is first produced to verify that the structure is self-supporting and also to serve as a guide for positioning the rattan rods along with the desired shape. Rattan serves as the framework in this fabrication approach since it is incorporated with the structural system and, as with all other components, is fully compostable. The number of reinforcement rods in the computed design is doubled to ensure the proper positioning of the hemp fiber and mycelium composite layers (Figure 9).

**Figure 10:**
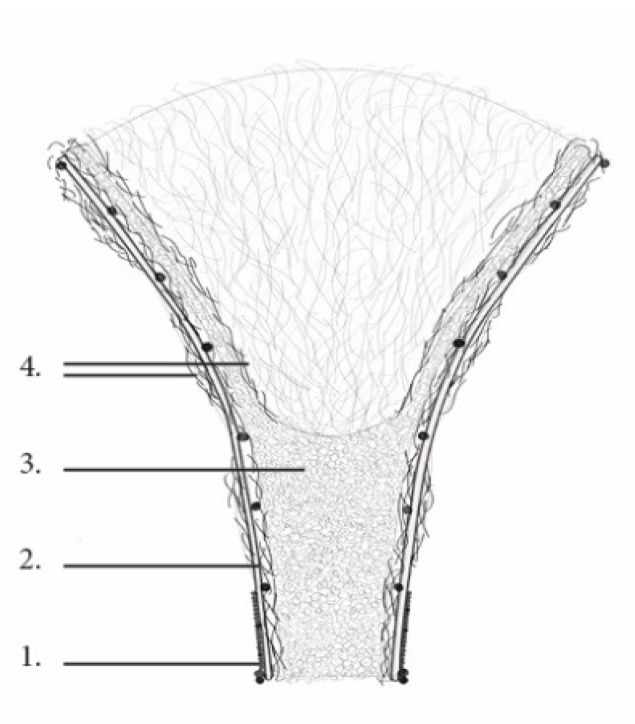
Section: 1. Rattan ⌀ 2 mm, 2. Rattan ⌀ 5 mm, 3. Mycelium substrate, 4. Hemp fibers.

Based on the results of Section 2.5, the rattan is the outer supporting skeleton; loose hemp fibers are flexible sub-layers for the mycelium to grow through and bind with the skeleton. Mycelium pre-grown substrate forms the core, which is subsequently covered with loose hemp fibers (Figure 10). Before assembly, fibers and rattan rods must be sterilized either by steaming or boiling. In addition, flour is added to the fibers to improve mycelium growth. Table 1 presents an overview of the materials used in the two experiments.

### 2.8. Assembly

**Figure 11:**
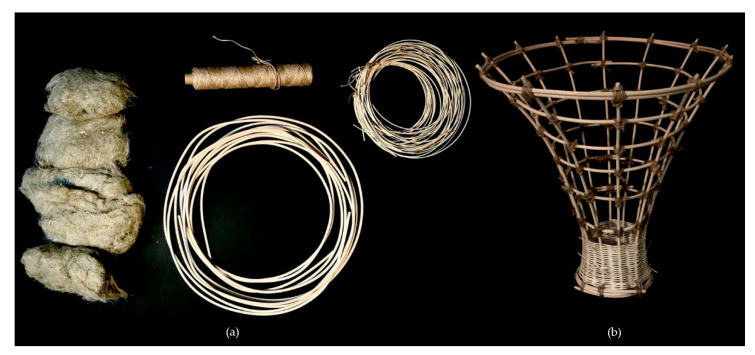
Material overview: (**a**) hemp fibers, rattan rods, jute fabric, (**b**) assembled rattan frame.

**Figure 12:**
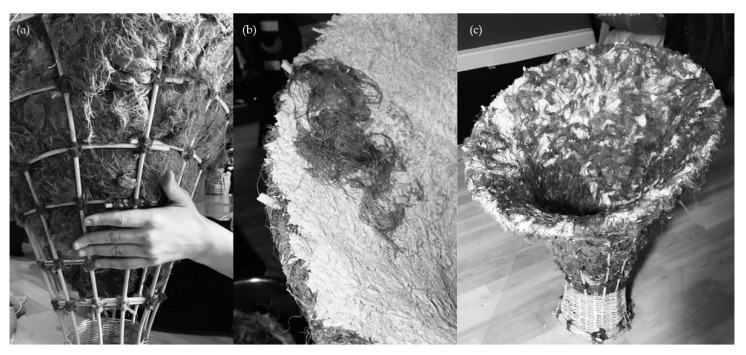
Assembly: (**a**), placing of fibers, (**b**) substrate and cover with additional layer of fibers, (**c**) assembled stool left to grow.

All materials must be sterilized before the assembly process begins. Figure 11a presents a material overview. After cooling down to room temperature, flour must be added to the wet hemp fibers. The rattan should be still wet so that one can bend the rods into their initial shape. The connection of vertical and horizontal members is secured with sterilized jute rope using traditional square knot techniques (Figure 11b). When the skeleton is assembled, the fibers can be placed on top so that no gaps emerge during the substrate placement (Figure 12a). This layer is approximately 1 cm thick. The pre-grown substrate is then mixed with psyllium husk until reaching a clay-like texture. Afterwards, the mixture is evenly distributed on top of the wet hemp fibers with a thickness of 3 cm (Figure 12b). An additional centimeter of wet hemp fibers follows. The multi-layer composite is then wrapped in perforated plastic foil to sustain moisture but also provide air circulation. To ensure constant moisture and nutritional levels, occasional spraying with a water-flour solution takes place. The assembled piece needs to be kept in a sterile environment for a minimum of 5 days while sufficient growth density can be reached (Figure 12c). Baking of the prototype at 80 degrees is then necessary to improve its compressive strength and to stop the growth process until the sample does not lose any further weight. While baking, a color change from white to a darker beige or brown is expected due to the hemp fibers.

## 3. Results

### 3.1. Comparison

Three scenarios are explored in order to determine the importance of including a rattan framework in the specified system:

#### 3.1.1. Composite without Rattan Reinforcement (Assumption)

Since a full prototype without a rattan skeleton was not created during this study, this scenario is an assumption. Although the soft mold samples indicate excellent merging capabilities with the mycelium-based core, the whole composite might not withstand the applied forces without the core breaking or splitting. A framework to fix the soft fabric to fill in the substrate, or an external mold, would be required to construct a composite without a rattan skeleton. During the growing and drying phases, the composite might deform unevenly due to the randomly oriented fibers.

#### 3.1.2. Composite with Rattan Reinforcement

The tensile capabilities of the mycelium-based rattan-reinforced composite have improved due to higher water content within the rods and wet hemp fibers, while the whole composite performs best under compression. Rattan’s load-bearing capacity is advantageous not only when merging with mycelium but also during the growing and drying phase to minimize uneven shrinking. The bottom support’s finely woven rattan maintains the core in place and prevents breakage.

#### 3.1.3. Rattan without Mycelium Matrix

**Figure 13:**
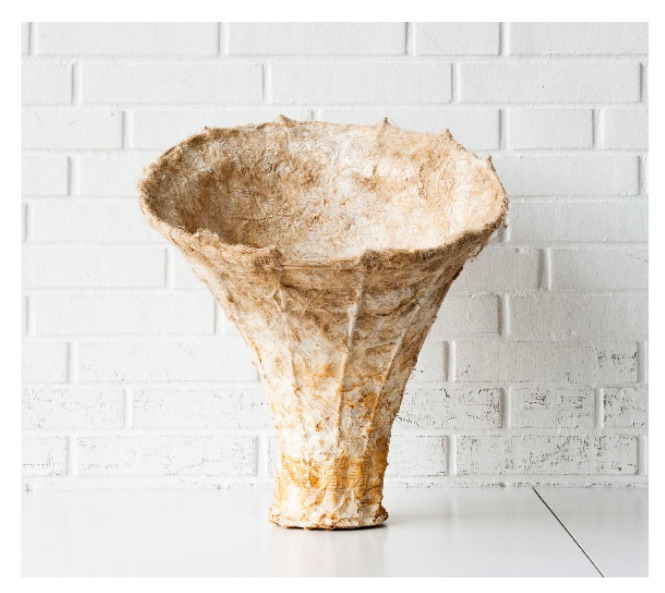
First Prototype.

Since rattan rods perform best under bending, this research demonstrated that rattan might be used as reinforcing rods in a mycelium composite. In the first attempt, the behavior of the rattan framework was tested through a seating test before placing fibers and substrate, which resulted in severe, irreversible deformation of the framework (Figure 13).

### 3.2. Physical Prototypes

**Figure 14:**
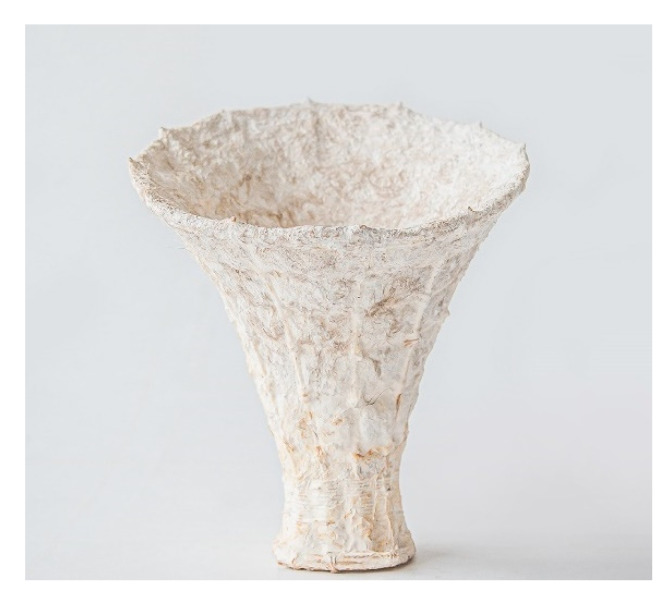
Final Prototype.

**Figure 15:**
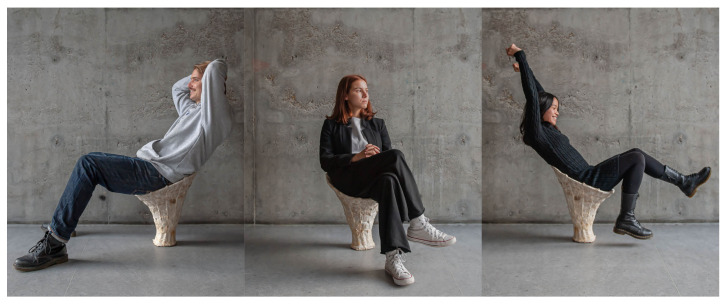
Seating tests (44 to 90 kilos).

In the first attempt, with an insufficient amount of 3 L of the substrate, the stool is able to hold the needed load but is still relatively unstable. The shell thickness varies from 0.5 cm to 1.5 cm. To improve its structural performance, the bottom radius is upscaled by 3 cm, which also prevents the stool from slipping. Additionally, in the second attempt, the amount of mycelium substrate is doubled, and 2.3 times more fibers are used. Psyllium husk is added to the substrate for improved material distribution, giving it a clay-like texture. In both attempts, mycelium binds effectively with all the elements. Significant growth has been observed in the vertical rattan members, particularly through the capillaries of the rattan. This is caused by the capillary effect, transporting the water, nutrients, and mycelium throughout the whole length. Due to increasing water content within the rods and wet hemp fibers, the tensile properties of the mycelium-based rattan-reinforced composite have improved, whereas the whole composite performs best under compression. In addition to rattan’s load-bearing capabilities when merging with mycelium, it is also beneficial in preventing uneven shrinking throughout the growing and drying process. In comparison to earlier research (Figure 1), in Mycomerge, the substrate entirely binds to the skin materials, leaving no evidence of separation. The densely woven rattan at the bottom support keeps the substrate in place and prevents breakage. The final prototype (Figure 14) weighs 3.7 kg and can support more than 20 times its own weight, demonstrating high structural capabilities and upscaling possibilities (Figure 15).

## 4. Architectural Application

**Figure 16:**
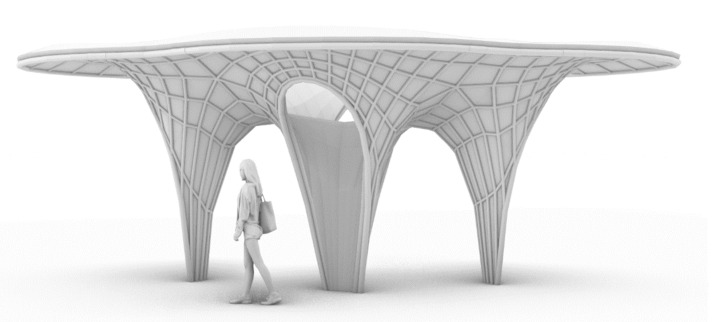
Architectural proposal.

Possible interior applications of the developed system can be in partition walls or sound insulation panels. Large elements can be fragmented, or the design can be developed in modular pieces able to fit in an industrial oven. In comparison with other fabrication techniques, working with rattan as a structural and form-giving framework eliminates the necessity of an external mold. Forming double-curved geometries can also be achieved. To showcase the potential of a full-scale structural application of the developed system, an initial design is developed (Figure 16). Since this structure is intended to be placed outside, it will not be baked, and the mycelium cultures will continue to grow in their natural environment until it decomposes. To prevent the growth of undesirable mold or other species, the growth phase must be interrupted, for instance, by exposing the structure to a high temperature of above 80 degrees or cooling it to below 0 degrees. Because an oven the size of an architectural building is unrealistic, assembly and growth are suggested to take place during the wintertime for the growth to stop naturally by simply being kept outside. However, this way of stopping the growing process may significantly influence the structural behavior and performance. Furthermore, this method is completely dependent on weather conditions and lacks consistency and applicability in various locations and seasons. The core material itself is not waterproof, and it will lose rigidity by being exposed to water and weathering. However, in between rainfalls, the material can dry and stabilize back again. By letting the structure air dry, mycelium can grow further on top of the surface, which will be covered with a pure mycelium layer. The foam-like mycelium layer does not absorb water, as one can see in several mycelium leather products found on the market. Keeping the structure fully waterproof is yet not possible, with an additional coating being necessary. The proposal of an exterior application is expected to last for two up to three months, similar to already developed mycelium temporary structures such as the Hy-Fi towers at MoMa in 2014. Further research on this topic is needed.

## 5. Discussion

Although the developed physical prototype has proven mycelium’s load-bearing and binding capacities, there is still a lot of space for further research on the structural capabilities of mycelium-based composites in large-scale applications. The necessity for interruption of the growth process presents limitations in the manufacturing on an architectural scale, due to the need for an industrial oven with a restricted size. That obstacle can be overcome through segmentation of the structural object into separate pre-grown and assembled on-site modules. For interior applications, Mycomerge presents a successful concept of material efficiency and load-bearing capacity of mycelium-based structures. Water content and moisture during growth are critical for the successful bonding of the rattan rods; otherwise, the mycelium will not grow onto the rattan’s surface. As an outcome, the tensile characteristics of the rattan rods and the mycelium composite will degrade, resulting in possible separation. In this study, rattan is utilized as an exterior skeleton; however, given the common reinforcement methods, such as steel rebar in concrete, more testing of layering, rattan binding, and reinforcement capabilities are required.

The materials used in this prototype are only agricultural waste products, which can be sourced regionally. Because all the components can be grown, this approach has no limitations in terms of resources. Mycomerge is fully biodegradable and hence promotes an eco-friendly alternative to the commonly used conventional materials, aiming toward sustainability in the building industry.

## Figures and Tables

**Table 1:** Material overview.

Material	First Prototype	Final Prototype
Hemp fibers (g)	180	420
Mycelium substrate (l)	3	7
Psyllium husk (g)	-	360
Jute rope (m)	100	100
Rattan (g) ⌀ 5 mm, 2 mm	250, 125	250, 125
Weight (kg)	2.1	3.7

## Data Availability

Not applicable.
